# The association between surgical duration and venous thromboembolism in outpatient surgery: A propensity score adjusted prospective cohort study

**DOI:** 10.1016/j.amsu.2020.11.003

**Published:** 2020-11-04

**Authors:** Kristi Pence, Daniel Fullin, Mark C. Kendall, Patricia Apruzzese, Gildasio De Oliveira

**Affiliations:** a Department of Anesthesiology, The Warren Alpert Medical School of Brown University, Providence, RI, USA; b Department of Anesthesiology, Rhode Island Hospital, Providence, RI, USA

**Keywords:** Outpatient surgery, Deep vein thrombosis, Pulmonary embolus, Surgery duration

## Abstract

**Background:**

Outpatient surgeries account for 60–70% of all procedures. Increased surgical duration has been demonstrated to be an independent risk factor for the development of venous thromboembolism (VTEs) after inpatient surgeries. In contrast, it is currently unknown if surgical duration increases the risk of VTEs for outpatient surgeries.

**Materials and methods:**

The 2005 through 2016 NSQIP Participant Use Data Files were queried to extract all patients scheduled for outpatient surgery. A z-score for surgical duration was calculated for each procedure to allow for standardization across surgeries of expected shorter or longer duration. The primary outcome measured was incidence of VTEs within 30 days of surgery.

**Results:**

A total of 3474 patients out of 1,863,523 (0.19%) had a VTE. After adjusting for confounding factors, the first and fifth quintiles compared to the middle quintile had odds ratios (ORs) of 0.75 (95% CI 0.68, 0.80) and 1.43 (95% CI, 1.35%–1.52%), respectively, *P* < 0.001. Patients who developed VTEs were more likely to be readmitted to the hospital, OR (95%CI) of 51.9 (48.0–56.2), C statistic = 0.67.

**Conclusion:**

Surgical duration is associated with the development of VTEs after outpatient surgery. While the overall incidence of VTE is low and does not require generalized prophylaxis, clinical practitioners should consider prophylaxis for patients undergoing outpatient surgery performed with excessive time compared to the average surgical procedure duration.

## Highlights

• Surgical duration is related with VTE development 30 days within outpatient surgery.
• Long outpatient surgeries resulted in a 1.43-fold increase in developing a VTE.
• Short outpatient surgeries resulted in a 25% reduction in developing a VTE.
• ENT surgery had the lowest VTE incidence while vascular surgery had the highest.
• Patients who developed VTEs were 52 times more likely to be readmitted.

## Graphical abstract

**Figure:**
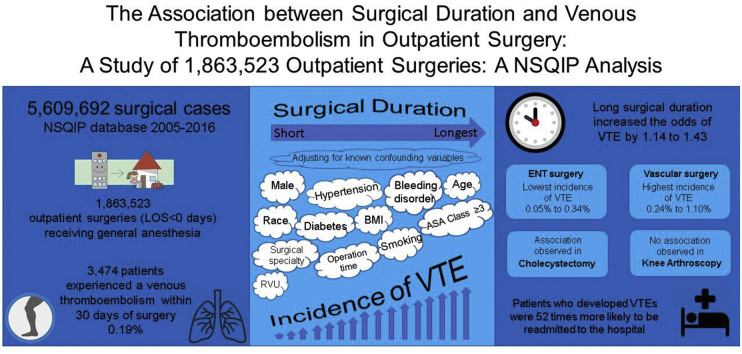

## 1 Introduction

Venous thromboembolism (VTE) is a significant cause of surgical morbidity and mortality with more than 500,000 hospitalizations and 100,000 deaths per year in the United States [1]. In addition, VTE prolongs hospital discharge and increases healthcare costs [2]. Drug prophylaxis with anticoagulants, compression stockings and early patient mobilization are the corner stones for the prevention of VTE [3]. Nonetheless, despite the wide implementation of prophylactic measures, VTE rates have not significantly improved over the last few years [4,5].

Outpatient surgeries account for 60–70% of all surgical procedures [6,7]. In addition, more complex surgeries (e.g., total knee arthroplasty, thyroidectomy, hysterectomy) with greater risks of VTEs are currently being performed in ambulatory facilities [[8], [9], [10]]. Increased surgical duration has been demonstrated to be an independent risk for the development of VTE after inpatient surgeries [11]. In contrast, it is currently unknown if surgical duration increases the risk of VTEs for outpatient surgeries. This knowledge would allow tailoring of VTE prophylaxis interventions to high risk patients undergoing outpatient surgery.

The main objective of the current investigation was to detect an independent association between surgical duration and VTE in patients undergoing outpatient surgery. We hypothesized that longer surgical procedures would be independently associated with greater rates of VTEs. We also sought to identify if the risk of VTEs varied according to specific outpatient surgical procedures.

## 2 Methods

This study was performed under an exempt status granted by the Institutional Review Board of Lifespan (IRB#1532635). The IRB determined that the study qualified for exemption under 45 CFR 46.101(b). The exemption was granted because the study involved a retrospective review of existing data recorded in such a manner that subjects cannot be identified, directly or through identifiers linked to the subjects. The study was registered at researchregistry.com (researchregistry5590). Clinical information of the subjects was obtained for the years 2005 through 2016 from the American College of Surgeons (ACS) National Surgical Quality Improvement Program (NSQIP) database. The study is reported following the STOBE and STROCSS guidelines for reporting cohort and observational studies [12,13].

The ACS NSQIP database is a national prospective database that compiles voluntarily reported data from over 680 institutions in the United States. Over 1 million cases were submitted as part of the 2016 update to the NSQIP database. Data is collected on over 300 variables that include preoperative risk factors, intraoperative variables and post-operative outcomes including complications up to 30 days after surgical procedures [14]. Data collection has been previously described in detail [14,15]. In brief, data is collected in 8-day cycles, with the first 40 procedures in the cycle included in the dataset. The most commonly performed procedures are capped at 5 within each cycle to increase procedure heterogeneity. Trained clinical nurses assigned at each site collected data for 30 days postoperatively using isolated telephone interviews and operative and clinical notes. Inter-rater reliability audits of selected participating sites help ensure the collected data is of the highest quality possible. The combined results of inter-rater reliability audits completed to date revealed an overall inter-rater disagreement rate of approximately 1.8% for all assessed program variables [16].

De-identified patient information is freely available to all institutional members who comply with the ACS NSQIP Data Use Agreement. The Data Use Agreement implements the protections afforded by the Health Insurance Portability and Accountability Act of 1996 and the. ACS NSQIP Hospital Participation Agreement. The ACS NSQIP and the hospitals participating in this program are the sources of the data used in this study; however, these entities have not been verified and are not responsible for the statistical validity of the data analysis or the conclusions derived by the authors.

The 2005 through 2016 NSQIP Participant Use Data Files were queried to extract all patients scheduled. Patients who qualified for the study under these criteria were then separated to an outpatient cohort, defined as length of stay (LOS) of 0 days. Cases described as cardiac and neurosurgery were excluded as those procedures are not routinely done in the outpatient setting. We also excluded trauma, fracture, neoplasms, infectious diseases or patients under 18 years of age. Surgical cases that were not performed under general anesthesia were omitted.

### 2.1 Primary explanatory and outcome variables

Total surgical duration was the primary independent variable examined. Surgical duration is measured from when surgery is first administered, until when the surgery is stopped. A z-score was calculated for each patient by dividing the difference between operative time and the mean surgical duration for the patient's CPT code by its standard deviation (SD). This allowed for standardization across surgeries of expected shorter or longer length. Cases were divided into five quintiles based on z-score, with the lowest quintile representing the shorter procedures. The primary outcome measured was incidence of venous thromboembolism within 30 days of surgery. Venous thromboembolism includes both deep vein thrombosis (DVT) and pulmonary embolus (PE). DVT is defined as an acute thrombus in the venous system and is diagnosed via ultrasound duplex, computed tomography (CT) or venogram. PE is diagnosed via CT, pulmonary arteriogram, or ventilation-perfusion scan. These definitions follow the definitions established by NSQIP.

### 2.2 Statistical analysis

Patient demographic data including age, sex, body mass index (BMI), race, and clinical characteristics such as inpatient status, American Society of Anesthesiologists class, smoking status, and surgical category were collected. Surgical categories included general, vascular, gynecologic, urologic, orthopedic, otolaryngologic (ENT), and plastic. Comorbidities were also collected, and diabetes mellitus, hypertension, and bleeding disorders were noted. The logarithm of the sum of relative value units (RVU) per procedure were used as a measure of surgical complexity. Missing data were analyzed for patterns and added using multiple imputation. Categorical variables were analyzed using Chi-square tests and continuous variables were analyzed using one-way analysis of variance tests. The Cochran-Armitage test for trend was used to assess the presence of a trend in surgical time and event rates.

Multiple logistic regression was used to determine the relationship between short (first and second quintiles) and long (fourth and fifth quintiles) operations and occurrence of VTE, relative to average length operations (middle quintile). A separate analysis examined the influence of the z score of the operative time as a continuous variable. To decrease the effect of uncommon CPT codes on the results, all CPT codes without a VTE and all CPT codes with fewer than 100 cases were discarded. A threshold of 0.05 was used for statistical significance, and all p-values were 2-sided. All statistical analyses were conducted with the use of SAS software version 9.4 (SAS Institute Inc., Cary, North Carolina).

## 3 Results

**Fig. 1:**
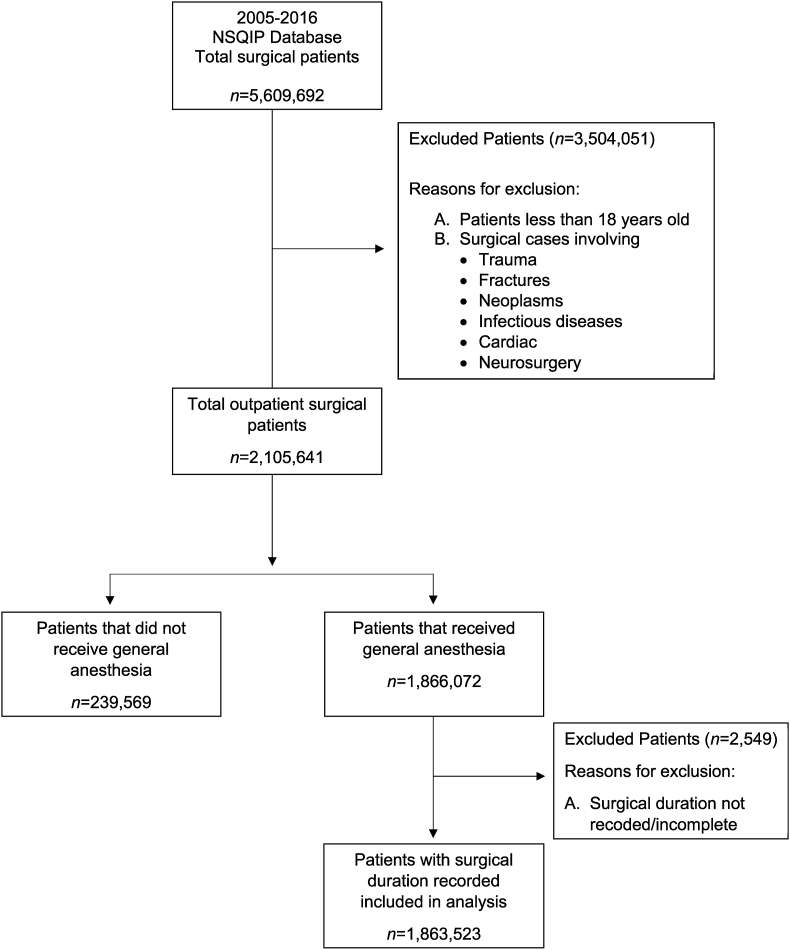
Flowchart of included and excluded cases.

The NSQIP database showed 5,286,671 surgical cases from 2005 to 2016. Of these, 2,105,641 patients were outpatients and 1,866,072 received general anesthesia for a specified time frame with 1,863,523 cases had non-missing surgical duration (Fig. 1). The patients were separated into 5 quintiles based on z score of their surgical duration. Age, BMI, sum relative value units, surgery time, sex, race, diabetes, smoking, hypertension, bleeding disorder and ASA class differed across quintiles and are presented in Table 1.

**Table 1:** Demographic and Preoperative Characteristics for Surgical Outpatients According to Five Quintiles Based on Z Score of their Surgical Duration.

Characteristic	1st Quintile [0 to 20th]	2nd Quintile [21st to 40th]	3rd Quintile [41st to 60th]	4th Quintile [61st to 80th]	5th Quintile [81st to 100th]	p-Value
**Age,** y, median (IQR)	52 (40, 64)	51 (38, 63)	51 (38, 63)	51 (39, 63)	52 (40, 63)	<.001
**Sex,** Male n (%)	139,021(37.3)	149,945 (40.3)	155,689 (41.8)	156,560(42.0)	159,127 (42.7)	<.001
**Race,** n (%)						
Caucasian	271,370 (87.1)	277,275 (85.7)	279,571 (84.5)	278,853 (83.3)	276,973 (82.0)	<.001
Black or AfricanAmerican	26,993 (8.7)	30,919 (9.6)	34,874 (10.5)	38,592 (11.5)	43,651 (12.9)	<.001
Asian	8,210 (2.6)	10,118 (3.1)	10,656 (3.2)	11,092 (3.3)	11,124 (3.3)	<.001
Other	5,119 (1.6)	5,206 (1.6)	5,673 (1.7)	6,099 (1.8)	6,039 (1.8)	<.001
**BMI**, kg/m^2^, median (IQR)	27.9 (24.3, 32.5)	28.3 (24.7, 33.1)	28.6 (24.9, 33.4)	28.9 (25.1, 33.8)	29.3 (25.4, 34.4)	<.001
**RVU**, median (IQR)	11.8 (8.0, 16.5)	10.5 (7.5, 15.6)	11.2 (8.0, 16.2)	11.9 (8.5, 17.1)	14.0 (9.5, 20.1)	<.001
**Operation time**, min, median (IQR)	31 (22, 46)	41 (28, 56)	53 (39, 72)	72 (57, 98)	118 (89, 168)	<.001
**Surgical Specialty, n (%)**						
General	223,934 (60.1)	226,313 (60.7)	224,451 (60.3)	223,913 (60.1)	218,249 (58.5)	<.001
Gynecology	35,228 (9.5)	29,734 (8.0)	29,103 (7.8)	30,720 (8.2)	35,087 (9.4)	<.001
Orthopaedic	48,295 (13.0)	54,801 (14.7)	55,002 (14.8)	53,012 (14.2)	50,962 (13.7)	<.001
ENT	15,013 (4.0)	18,434 (4.9)	19,800 (5.3)	18,680 (5.0)	18,575 (5.0)	<.001
Plastics	20,870 (5.6)	14,827 (4.0)	15,265 (4.1)	16,827 (4.5)	20,358 (5.5)	<.001
Urology	18,993 (5.1)	20,820 (5.6)	20,504 (5.5)	20,067 (5.4)	19,181 (5.2)	<.001
Vascular	1,0379 (2.8)	7,891 (2.1)	8,433 (2.3)	9,532 (2.6)	10,270 (2.8)	<.001
**Diabetes mellitus, n (%)**	37,404 (10.0)	37,419 (10.0)	38,305 (10.3)	39,908 (10.7)	41,868 (11.2)	<.001
**Smoking, n (%)**	68,547 (18.4)	67,058 (18.0)	65,988 (17.7)	64,855 (17.4)	64,197 (17.2)	<.001
**Hypertension, n (%)**	127,268 (34.2)	125,018 (33.5)	125,966 (33.8)	129,573 (34.8)	134,463 (36.1)	<.001
**Bleeding disorder, n (%)**	6,280 (1.7)	5,823 (1.6)	5,877 (1.6)	5,803 (1.6)	5,867 (1.6)	<.001
**ASA class ≥ 3, n (%)**	95,481 (25.7)	95,844 (25.7)	98,410 (26.4)	104,028 (27.9)	111,398 (29.9)	<.001

ASA class = American Society of Anesthesiologists classification; BMI = body mass index; IQR = interquartile range; RVU = relative value units.

**Fig. 2:**
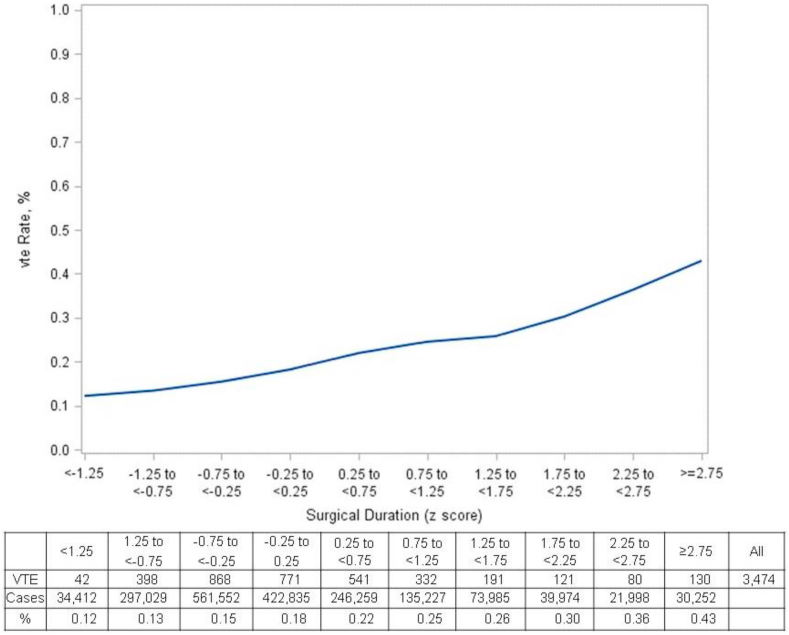
The Influence of Surgical Duration on Venous Thromboembolism Rates in Outpatient Surgery**.** The relationship between the z-score of surgical duration and the incidence of venous thromboembolism is shown. This figure details the rates of venous thromboembolism at each point estimate.

A total of 3,474 patients (0.19%) had a VTE, 2341 (0.13%) experienced a DVT, 1,450 (0.08%) developed a PE, and 317 (0.02%) experienced both a DVT and PE. The rates of VTE, DVT and PE increased as surgical duration increased (Fig. 2).

The middle quintile of procedures showed a VTE rate of 0.17%. Compared to the middle quintile, the first and second quintiles had odds ratios (ORs) of 0.74 (0.68, 0.80) and 0.87 (95% CI, 0.81 to 0.94), respectively, *P* < 0.001. The fourth and fifth quintiles demonstrated ORs of 1.14 (1.07, 1.22) and 1.43 (95% CI, 1.35 to 1.52), respectively, *P* < 0.001 (Table 2). A sensitivity analysis demonstrated that the odds of VTE increase as surgical duration increases when considering surgical duration as a continuous variable after adjusting for confounding factors.

**Table 2:** Risk adjusted model for venous thromboembolism across different quintiles of surgical duration.

Quintile	Events (#)	Unadjusted	Adjusted			
		Events (%)	Proportion, % (95% CI)	Risk Difference	OR (95% CI)	p-Value
1st [0 to 20th]	497	0.13	0.142 (0.129, 0.157)	−0.04 (−0.06, −0.02)	0.74 (0.68, 0.80)	<.0001
2nd [21st to 40th]	565	0.15	0.167 (0.153, 0.184)	−0.02 (−0.04, 0.01)	0.87 (0.81, 0.94)	0.0002
3rd [41st to 60th]	628	0.17	0.183 (0.168, 0.20)	Reference	Reference	–
4th [61st to 80th]	777	0.21	0.22 (0.202, 0.238)	0.04 (0.01, 0.06)	1.14 (1.07, 1.22)	<.0001
5th [81st to 100th]	1,007	0.27	0.276 (0.256, 0.297)	0.09 (0.07, 0.12)	1.43 (1.35, 1.52)	<.0001
Z Score	.				1.15 (1.13, 1.17)	<.0001

Adjusted for sex, surgical specialty, diabetes mellitus, smoking, hypertension, bleeding disorder, American Society of Anesthesiologists classification, body mass index and relative value units. C statistic = 0.694 (95% CI: 0.685, −0.703), Cochran-Armitage Trend Test P < 0.001.

**Fig. 3:**
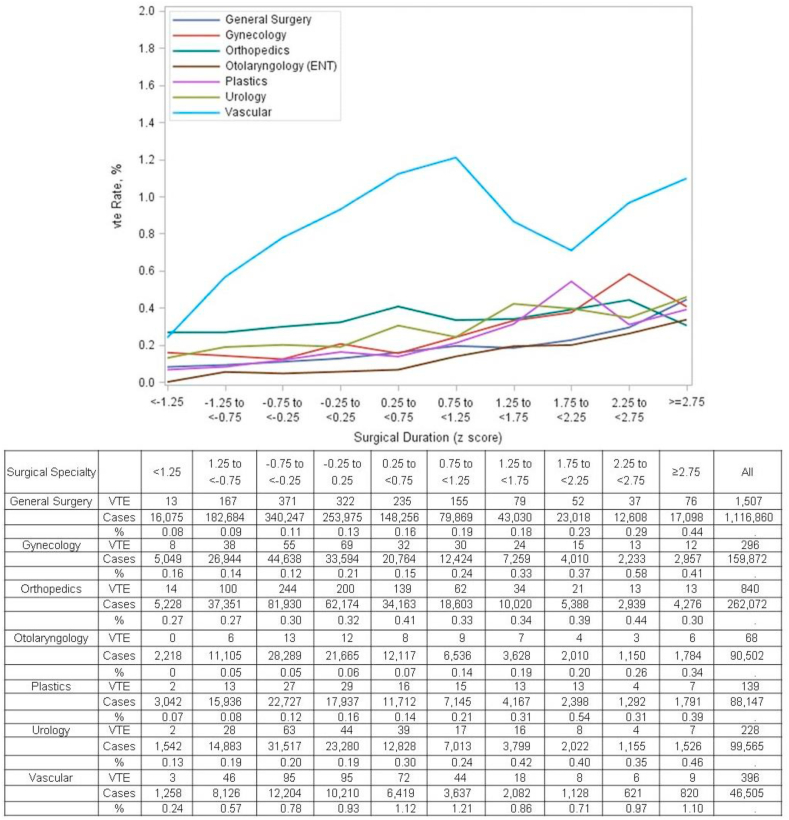
The Influence of Surgical Duration on Venous Thromboembolism Rates by Surgical Specialty. The relationship between the z-score of surgical duration and the incidence of venous thromboembolism is shown by surgical specialties. This figure details the rates of venous thromboembolism at each point estimate for each surgical specialty.

The odds ratio for each unit of surgical time was significant for all surgical specialties except orthopedics in the subgroup analysis (Fig. 3). The VTE rate for specialties differed with ENT having the lowest incidence (0.05% in shortest procedures and 0.34% in longest procedures). Vascular surgery had the highest incidence of VTE (0.24% in shortest procedures and 1.10% in longest procedures).

We also examined the association of VTE with surgical duration in common outpatient procedures across different specialties. We found that the association is likely procedure specific (Table 3). For example, VTE was associated with surgical duration for patients undergoing cholecystectomy, OR (95%CI) of 1.29 (1.44–2.26), while no association was detected for knee arthroscopy, OR (95%CI) of 1.08 (0.81–1.44).

**Table 3:** Risk adjusted analysis of venous thromboembolism by surgical duration for common outpatient surgical procedures across specialties.

Surgical Procedures	Cases (n)	Events (n)	Surgical time, min mean ± SD	OR (95% CI), per min	OR (95% CI), per hour	OR (95% CI), per SD	p-Value
Cholecystectomy	163,600	189	56.98 ± 31.75	1.00 (1.00, 1.01)	1.30 (1.17, 1.44)	1.23 (1.13, 1.33)	<.001^†^
Total Hysterectomy	38,434	104	127.97 ± 57.59	1.00 (1.00, 1.01)	1.31 (1.14, 1.50)	1.36 (1.16, 1.59)	<.001^†^
Knee Arthroscopy	42,285	175	31.49 ± 25.29	1.00 (0.99, 1.00)	1.08 (0.82, 1.44)	1.07 (0.85, 1.34)	0.577
Tonsillectomy	21,325	4	27.32 ± 27.06	1.01 (1.00, 1.01)	1.43 (1.01, 2.02)	1.50 (1.01, 2.23)	0.044^†^
Reduction Mammaplasty	20,086	31	152.25 ± 68.61	1.00 (1.00, 1.01)	1.27 (1.02, 1.58)	1.37 (1.02, 1.84)	0.034^†^
Varicose Vein	4,930	45	62.45 ± 33.73	1.01 (1.00, 1.02)	1.78 (1.17, 2.73)	1.65 (1.14, 2.39)	0.008^†^
Scope Bladder Removal of Tumors	11,605	16	24.19 ± 20.92	1.00 (0.99, 1.02)	1.34 (0.69, 2.62)	1.30 (0.71, 2.38)	0.389

Adjusted for sex, diabetes mellitus, hypertension, bleeding disorder, American Society of Anesthesiologists classification, body mass index and relative value units. CI = confidence interval; OR = odds ratio; SD = standard deviation. † = Statistically significant.

VTEs had an important role on hospital readmission. After adjusting for potential confounding factors (sex, surgical specialty, diabetes mellitus, smoking, hypertension, bleeding disorder, American Society of Anesthesiologists class ≥3, body mass index, and RVU), patients who developed VTEs had a greater propensity for hospital readmissions, OR (95%CI) of 51.9 (48.0–56.2), C statistic = 0.67.

## 4 Discussion

The most important finding of the current study was the direct association between surgical duration and the rate of venous thromboembolism within 30 days of outpatient surgery. After adjusting for potential confounding factors, outpatient procedures that were classified in the top quintile of surgery duration had a 1.43-fold increase in the odds of developing a VTE compared with procedures of average duration. In contrast, outpatient procedures that were classified in the bottom quintile of surgery duration had a 25% reduction on VTE. Taken together, our results suggest that the reduction of surgical duration should be an important goal in order to decrease thromboembolic complications after outpatient surgery.

Our findings are clinically important as outpatient surgery is often perceived as low risk and patients are often expected to provide self-care over a short period of time. VTE is an important cause of morbidity and mortality after surgery. VTE is particularly problematic after outpatient surgery where patients do not have hospital support to help with their postoperative care. Surgical duration can be used to tailor VTE prophylaxis to the highest risk patients. To the best of our knowledge, this is the first study to examine the relationship between surgical duration and VTEs in outpatient surgery.

Another important finding of the current investigation was the fact that the association between VTE and surgical duration was procedure specific. The association was present for patients undergoing cholecystectomy or reduction mammoplasty, but it was not present for patients undergoing knee arthroscopy and scope bladder removal of tumors. The lack of association between surgical duration and VTE in some specific procedures was mainly observed in short duration procedures (e.g., mean duration < 30 min), but not in longer procedures (e.g. mean surgical duration ≥30 min).

One may argue that surgical duration is a difficult variable to reduce as it is often related to surgical complexity. We addressed the surgical complexity by not only accounting for patient factors, but also included surgical RVUs in the multivariate model. Prior studies have demonstrated that RVUs is a validated metric for surgical complexity [17,18]. Surgical duration also varies among different procedures across specialties. We addressed this fact by creating a z-score for each surgery to normalize the variability of surgical duration for each specific procedure.

A prior investigation has evaluated the effect of surgery duration and venous thromboembolism in patients undergoing inpatient surgery [11]. We observed a lower incidence of VTEs after outpatient surgery (0.19%) compared to the prior study in inpatient surgery (0.96%). Nonetheless, we detected a stronger association of surgical duration and VTE after outpatient surgery (1.42-fold) versus the prior study examining inpatient surgery (1.27-fold). Patients undergoing inpatient surgery have more comorbidities (e.g. cancer) that can reduce the magnitude of the association between surgical duration and postoperative VTEs.

Our study should be considered in the context of its limitations. Using data from the NSQIP database allowed us to control for many patient and surgical variables. However, the database did not include some information, such as chemoprophylactic measures, which limited our ability to control for these variables. Nonetheless, since patients are expected to ambulate after outpatient surgery, the risk of VTE is considered small to warrant chemoprophylaxis. Lastly, the nature of an observational study design does not allow us to demonstrate that increased surgical duration causes VTE events. However, quantifying the strength of the association between surgical duration and VTE for various types of outpatient procedures provides useful information to direct further studies and hospital policies.

## 5 Conclusions

In summary, we have demonstrated that surgical duration is associated with the development of VTEs after ambulatory surgery. While the overall incidence of VTE is low after ambulatory surgery and does not require generalized prophylaxis, clinical practitioners should consider prophylaxis for patients undergoing outpatient surgery performed with excessive time compared to the average surgical procedure duration.

## Financial source

This research did not receive any specific grant from funding agencies in the public, commercial, or not-for-profit sectors.

## Provenance and peer review

Not commissioned, externally peer reviewed.

## Declaration of competing interest

None.
